# Supplementary material for: Branch point strength controls species-specific CAMK2B alternative splicing and regulates LTP
Source: Life Sci Alliance. 2022 Dec 21;6(3):e202201826. doi: 10.26508/lsa.202201826 (PMC9772828; doi:10.26508/lsa.202201826)
Supplement: Supplementary file 3 [file LSA-2022-01826_TableS1.docx]

| Supplementary Table 1. CaMKIIβ autophosphorylation sites are isoform-specific. |
| --- |
| \|  \| **Log2 (Enrichment)** \| \| \| \| \|  \| \|  \| \| --- \| --- \| --- \| --- \| --- \| --- \| --- \| --- \| --- \| \| **Unique phospho-site** \| **FL/Ctr** \| **13/Ctr** \| **16,17/**  **Ctr** \| **13,16/**  **Ctr** \| **Location of phosphosite** \| \| **Function** \| \| \| \| **Camk2b-S315** \| unique \| absent \| unique \| absent \| Exon 12 \| \| F-actin binding ([Kim et al., 2015](#_ENREF_40)) \| \| \| \| **Camk2b-T320** \| unique \| absent \| unique \| absent \| Exon 13 \| \| F-actin binding ([Kim et al., 2015](#_ENREF_40)) \| \| \| \| **Camk2b-T321** \| unique \| absent \| unique \| absent \| Exon 13 \| \| F-actin binding ([Kim et al., 2015](#_ENREF_40)) \| \| \| \| **Camk2a-T306** \| unique \| absent \| unique \| absent \| Exon 12 \| \| Inactivating ([Colbran and Soderling, 1990](#_ENREF_13)) \| \| \| \| **Camk2a-T307** \| unique \| unique \| unique \| unique \| Exon 12 \| \| Inactivating ([Colbran and Soderling, 1990](#_ENREF_13)) \| \| \| \| **Camk2b-S534** \| 1,17 \| 0,95 \| -0,51 \| -0,81 \| Hub Domain \| \| NA \| \| \| \| **Camk2b-S280** \| 5,68 \| 5,58 \| 4,58 \| 4,98 \| Exon 11 \| \| GlcNAc site ([Erickson et al., 2013](#_ENREF_19)) \| \| \| \| **Camk2b-T287** \| 6,57 \| 6,48 \| 5,47 \| 5,88 \| Exon 11 \| \| Activating ([Miller et al., 1988](#_ENREF_49)) \| \| \| \| **Camk2d-S280** \| 2,74 \| 2,47 \| 1,58 \| absent \| Exon 11 \| \| GlcNAc site ([Erickson et al., 2013](#_ENREF_19)) \| \| \| \| **Camk2d-T287** \| 2,74 \| 2,47 \| 1,58 \| absent \| Exon 11 \| \| Activating \| \| \| \| **Camk2b-T254** \| unique \| unique \| unique \| unique \| Kinase Domain \| \| NA \| \| \| \| **Camk2b-S276** \| unique \| unique \| absent \| unique \| Exon 11 \| \| NA \| \| \| \| **Camk2b-S71** \| absent \| unique \| absent \| unique \| Kinase Domain \| \| NA \| \| \| |
| Supplementary Table S1. The list of detected phosphorylation sites was restricted to gene names CaMK2a, CaMK2b, CaMK2d. Numbers are Log2 ratio between the average intensity values for this isoform vs. controls. Unique: this phosphorylation target was not detected in the corresponding control samples (UT and K43R) and thus no Log2 ratio could be calculated. Absent: this phosphorylation site was not detected in the corresponding sample. |

**References**

Colbran, R.J., and Soderling, T.R. (1990). Calcium/calmodulin-independent autophosphorylation sites of calcium/calmodulin-dependent protein kinase II. Studies on the effect of phosphorylation of threonine 305/306 and serine 314 on calmodulin binding using synthetic peptides. Journal of Biological Chemistry 265, 11213-11219.

Erickson, J.R., Pereira, L., Wang, L., Han, G., Ferguson, A., Dao, K., Copeland, R.J., Despa, F., Hart, G.W., Ripplinger, C.M.*, et al.* (2013). Diabetic hyperglycaemia activates CaMKII and arrhythmias by O-linked glycosylation. Nature *502*, 372-376.

Kim, K., Lakhanpal, G., Lu, Hsiangmin E., Khan, M., Suzuki, A., Kato Hayashi, M., Narayanan, R., Luyben, Thomas T., Matsuda, T., Nagai, T.*, et al.* (2015). A Temporary Gating of Actin Remodeling during Synaptic Plasticity Consists of the Interplay between the Kinase and Structural Functions of CaMKII. Neuron *87*, 813-826.

Miller, S.G., Patton, B.L., and Kennedy, M.B. (1988). Sequences of autophosphorylation sites in neuronal type II CaM kinase that control Ca2+-independent activity. Neuron *1*, 593-604.
