# Supplementary material for: Branch point strength controls species-specific CAMK2B alternative splicing and regulates LTP
Source: Life Sci Alliance. 2022 Dec 21;6(3):e202201826. doi: 10.26508/lsa.202201826 (PMC9772828; doi:10.26508/lsa.202201826)
Supplement: Supplementary file 4 [file LSA-2022-01826_TableS2.docx]

| Supplementary Table S2. Publicly available RNA-Seq datasets used in this study. |
| --- |
| \| SRA \| Species \| Tissue \| Read length \| paired/single \| Reference Genome \| \| --- \| --- \| --- \| --- \| --- \| --- \| \| SRR8750487 \| *Human* \| Cerebellar White Matter \| 150 bp \| paired-end \| GRCh38 \| \| SRR8750488 \| *Human* \| Cerebellar White Matter \| 150 bp \| paired-end \| GRCh38 \| \| SRR8750489 \| *Human* \| Cerebellar White Matter \| 150 bp \| paired-end \| GRCh38 \| \| SRR8750490 \| *Human* \| Cerebellar White Matter \| 150 bp \| paired-end \| GRCh38 \| \| SRR8750491 \| *Human* \| Cerebellar Grey Matter \| 150 bp \| paired-end \| GRCh38 \| \| SRR8750492 \| *Human* \| Cerebellar Grey Matter \| 150 bp \| paired-end \| GRCh38 \| \| SRR8750493 \| *Human* \| Cerebellar Grey Matter \| 150 bp \| paired-end \| GRCh38 \| \| SRR8750647 \| *Pan troglodytes* \| Cerebellar White Matter \| 150 bp \| paired-end \| panTro6 \| \| SRR8750679 \| *Pan troglodytes* \| Cerebellar White Matter \| 150 bp \| paired-end \| panTro6 \| \| SRR8750711 \| *Pan troglodytes* \| Cerebellar White Matter \| 150 bp \| paired-end \| panTro6 \| \| SRR8750648 \| *Pan troglodytes* \| Cerebellar Grey Matter \| 150 bp \| paired-end \| panTro6 \| \| SRR8750680 \| *Pan troglodytes* \| Cerebellar Grey Matter \| 150 bp \| paired-end \| panTro6 \| \| SRR8750712 \| *Pan troglodytes* \| Cerebellar Grey Matter \| 150 bp \| paired-end \| panTro6 \| \| SRR8750448 \| *Pan paniscus* \| Cerebellar White Matter \| 150 bp \| paired-end \| panPan1.1 \| \| SRR8750449 \| *Pan paniscus* \| Cerebellar White Matter \| 150 bp \| paired-end \| panPan1.1 \| \| SRR8750450 \| *Pan paniscus* \| Cerebellar White Matter \| 150 bp \| paired-end \| panPan1.1 \| \| SRR8750451 \| *Pan paniscus* \| Cerebellar Grey Matter \| 150 bp \| paired-end \| panPan1.1 \| \| SRR8750452 \| *Pan paniscus* \| Cerebellar Grey Matter \| 150 bp \| paired-end \| panPan1.1 \| \| SRR8750595 \| *Pan paniscus* \| Cerebellar Grey Matter \| 150 bp \| paired-end \| panPan1.1 \| \| SRR5804509 \| *Gorilla gorilla* \| Cerebellum \| 101 bp \| paired-end \| gorGor6 \| \| SRR5804501 \| *Gorilla gorilla* \| Cerebellum \| 101 bp \| paired-end \| gorGor6 \| \| SRR306801 \| *Gorilla gorilla* \| Brain \| 101 bp \| paired-end \| gorGor6 \| \| SRR10393301 \| *Pongo pygmaeus abelii* \| Testis \| 150 bp \| paired-end \| ponAbe3 \| \| SRR10393303 \| *Pongo pygmaeus abelii* \| Testis \| 150 bp \| paired-end \| ponAbe3 \| \| SRR10393302 \| *Pongo pygmaeus abelii* \| Testis \| 150 bp \| paired-end \| ponAbe3 \| \| SRR10393304 \| *Pongo pygmaeus abelii* \| Testis \| 150 bp \| paired-end \| ponAbe3 \| \| DRR128395 \| *Pongo pygmaeus* \| Skin \| 125 bp \| paired-end \| ponAbe3 \| \| DRR128394 \| *Pongo pygmaeus* \| Skin \| 100 bp \| paired-end \| ponAbe3 \| \| DRR128393 \| *Pongo pygmaeus* \| Skin \| 100 bp \| paired-end \| ponAbe3 \| \| SRR306792 \| *Pongo pygmaeus* \| Brain \| 150 bp \| paired-end \| ponAbe3 \| \| SRR5804517 \| *Hylobates lar* \| Cerebellum \| 100 bp \| paired-end \| nomLeu3 (*Nomascus leucogenys*) \| \| SRR5804510 \| *Hylobates lar* \| Dorsolateral Prefrontal Cortex \| 100 bp \| paired-end \| nomLeu3 (*Nomascus leucogenys*) \| \| SRR5804511 \| *Hylobates lar* \| Ventrolateral Prefrontal Cortex \| 100 bp \| paired-end \| nomLeu3 (*Nomascus leucogenys*) \| \| SRR5804512 \| *Hylobates lar* \| Premotor Cortex \| 100 bp \| paired-end \| nomLeu3 (*Nomascus leucogenys*) \| \| SRR5804513 \| *Hylobates lar* \| Primary Visual Cortex \| 100 bp \| paired-end \| nomLeu3 (*Nomascus leucogenys*) \| \| SRR5804514 \| *Hylobates lar* \| Anterior Cingulate Cortex \| 100 bp \| paired-end \| nomLeu3 (*Nomascus leucogenys*) \| \| SRR5804515 \| *Hylobates lar* \| Striatum \| 100 bp \| paired-end \| nomLeu3 (*Nomascus leucogenys*) \| \| SRR5804516 \| *Hylobates lar* \| Hippocampus \| 100 bp \| paired-end \| nomLeu3 (*Nomascus leucogenys*) \| \| SRR8750552 \| *Macaca mulatta* \| Cerebellar White Matter \| 150 bp \| paired-end \| rheMac10 \| \| SRR8750553 \| *Macaca mulatta* \| Cerebellar White Matter \| 150 bp \| paired-end \| rheMac10 \| \| SRR8750554 \| *Macaca mulatta* \| Cerebellar White Matter \| 150 bp \| paired-end \| rheMac10 \| \| SRR8750549 \| *Macaca mulatta* \| Cerebellar Grey Matter \| 150 bp \| paired-end \| rheMac10 \| \| SRR8750550 \| *Macaca mulatta* \| Cerebellar Grey Matter \| 150 bp \| paired-end \| rheMac10 \| \| SRR8750551 \| *Macaca mulatta* \| Cerebellar Grey Matter \| 150 bp \| paired-end \| rheMac10 \| \| SRR11939284 \| *Sus scrofa* \| Cerebellum \| 150 bp \| paired-end \| SusScr11 \| \| SRR11939285 \| *Sus scrofa* \| Cerebellum \| 150 bp \| paired-end \| SusScr11 \| \| SRR11939286 \| *Sus scrofa* \| Cerebellum \| 150 bp \| paired-end \| SusScr11 \| |
